# The introduction of Greek Central Health Fund: Has the reform met its goal in the sector of Primary Health Care or is there a new model needed?

**DOI:** 10.1186/s12913-014-0583-4

**Published:** 2014-11-25

**Authors:** Nikos Polyzos, Stefanos Karakolias, Costas Dikeos, Mamas Theodorou, Catherine Kastanioti, Kalomira Mama, Periklis Polizoidis, Christoforos Skamnakis, Charalampos Tsairidis, Eleutherios Thireos

**Affiliations:** Department of Social Administration and Political Science, Democritus University of Thrace, Komotini, Greece; Faculty of Economics and Management, Open University of Cyprus, Nicosia, Cyprus; Department of Management of Enterprises and Organizations, ATEI of Peloponnese, Kalamata, Greece; Health Center of Vari, Vari, Greece

**Keywords:** Health systems, Health funds, EOPYY, Primary health care, Reimbursement, APGs

## Abstract

**Background:**

The National Organization for Healthcare Provision (EOPYY) originates from the recent reform in Greek healthcare, aiming amidst economic predicament, at the rationalization of health expenditure and reactivation of the pivotal role of Primary Health Care (PHC). Health funding (public/private) mix is examined, alongside the role of pre-existing health insurance funds. The main pursuit of this paper is to evaluate whether EOPYY has met its goals.

**Methods:**

The article surveys for best practices in advanced health systems and similar sickness funds. The main benchmarks focus on PHC provision and providers’ reimbursement. It then turns to an analysis of EOPYY, focusing on specific questions and searching the relevant databases. It compares the best practice examples to the EOPYY (alongside further developments set by new legislation in L 4238/14), revealing weaknesses relevant to non-integrated PHC network, unbalanced manpower, non-gatekeeping, under-financing and other funding problems caused by the current crisis. Finally, a new model of medical procedures cost accounting was tested in health centers.

**Results:**

An alternative operation of EOPYY functioning primarily as an insurer whereas its proprietary units are integrated with these of the NHS is proposed. The paper claims it is critical to revise the current induced demand favorable reimbursement system, via per capita payments for physicians combined with extra pay-for-performance payments, while cost accounting corroborates a prospective system for NHS’s and EOPYY’s units, under a combination of global budgets and Ambulatory Patient Groups (APGs)

**Conclusions:**

Self-critical points on the limitations of results due to lack of adequate data (not) given by EOPYY are initially raised. Then the issue concerning the debate between ‘copying’ benchmarks and ‘a la cart’ selectively adopting and adapting best practices from wider experience is discussed, with preference to the latter. The idea of an ‘a la cart’ choice of international examples is proposed. The ‘results’ discussing EOPYY’s dual function and induced-demand favorable reimbursement system are further critically examined. International experience shows evidence of effective alternatives, such as per capita and pay-for-performance payments for practicing doctors as well as per case reimbursement for health centers under global budget principles.

## Background

Current challenging economic climate prompts many governments in and beyond the European Union to work towards increasing efficiency and curbing expenditure in health systems. The Greek health system is a mixture of (a) public integrated, (b) public contract and (c) public reimbursement models, comprising elements from both the public and private sectors and incorporating principles of different organizational patterns. The system is therefore financed by state budget, social insurance contributions and private payments. Taxation contributes 29.1% of total health expenditure, while health insurance accounts for 31.2%. It should be noted that private expenditure amounts for a very high percentage of the mixed financial resources, and this public/private mixture is a significant feature of the system. In a little more detail, out-of-pocket payments account for 37.6% of total health expenditure, whilst private insurance accounts for 2.1%, calling the social character of the health system into question [1]. However, total (public and private) health expenditure has reduced since 2010 financial crisis (from almost 10 to 8% of GDP).

Further to the long standing existence of numerous social and health insurance funds, of compulsory participation, with the 2011 health insurance reforms resulting to a unified central health fund (EOPYY: National Organization for Healthcare Provision), the Greek Government attempted to minimize the burden on the state budget of subsidizing larger and troublesome funds by transferring funds from these that are financially better off [2] to these that are in a less favorable position. Although EOPYY’s establishment is undoubtedly the most promising reform of the last decades in Greek health insurance, its performance doesn’t seem to have met the expectations of the Greek Government. The organization is engaged in a vicious circle of deficits, although declining from 2.5 billion euros in 2012 to 1.2 billion euros in 2013, which generally characterize the domestic social insurance system [3].

Primary Health Care (PHC) is a key factor in contemporary health systems acting both as a point of first contact and a gatekeeping mechanism. PHC in Greece was short while ago provided by both NHS and EOPYY units however a large number of self-employed health professionals still exist. More specifically, PHC relies on health centers and private or public hospitals’ outpatient clinics, assigned to the NHS; EOPYY’s polyclinics and medical offices; and physicians, nurses, pharmacists, physiotherapists and other self-employed health professionals contracted with the EOPYY. The current scheme allows the free choice of provider but free choice of insurer is prohibited. Structurally, Greek general practitioners (GPs) are under-numbered (compared to specialists), there are a few nurses per thousand of population, and urban areas attract most providers and patients. As a result, gatekeeping and family physician institutions do not exist, letting patients prefer the option of using secondary care structures.

This paper puts forward a proposal for reforming the structure of Greek PHC and reimbursement system for PHC providers contracted with EOPYY, undertaken by a research project conducted by a multidisciplinary team of both academic staff and individuals during the period November 2012 - July 2013 and overseen by the Special Account for Research Grants of Democritus University of Thrace.

## Methods

The methodology includes a selective review of healthcare purchasing and provision systems in developed western countries, and an analysis of the current situation of the Greek PHC and EOPYY, as to point out both best practices (or indeed poor examples to be avoided) and problems/shortcomings in the Greek case to be tackled. The study of health systems highlights issues, such as organizational structure, operation and coverage, and last but not least financing and remuneration methods. This spectrum of systems and funds includes the state centered Anglo-Saxon (“Beveridge”) tax-based system providing universal access and coverage; the continental (“Bismarck”) model financed by social insurance; and corporate elements from the private model [4]. A brief summary of some of the most important findings that are taken into account in our research follows in the next subsection "A selective overview of health systems", leaving subsection "Overview and analysis of Greek Healthcare focusing upon the EOPYY and PHC" for a more detailed examination of the Greek case and "Cost accounting under APGs principles" for setting a standard costing procedure.

### a. A selective overview of health systems

We have chosen to focus upon eight different and well-developed health systems.

Three of them are taxation-funded, universal and compulsory: the British - or indeed English as minor deviations exist in Scotland - that is considered an archetype and now operates through contractual agreements between 151 commissioning healthcare organizations and healthcare practices operated by GPs [5]; the Swedish that is organized and managed on three levels: national, regional and municipal and promotes equality in access on the base of a relatively *de-commodified* (despite current rationalization) provision of social and health services as access to PHC is not free of charge, whereas there are provisions for maximum cost per service [6-10]; and the Spanish that operates upon the regional structure of the country, with each region having a ministry of health, and strict separation alongside good co-operation of (between) primary and special care, giving access to all population including a significant part of (illegal) migrants [11,12].

Five systems are insurance based, four of them compulsory of which three are public (Germany [13,14], Austria [15] and France [16-18]) administered by autonomous insurance funds (Germany and Austria, with Germany moving towards unification and centralization through AOK since 2009), or a unified state fund (France), alongside federal structures of the states themselves (Germany, Austria) purchasing services from private providers who in the case of PHC act as gatekeepers too, and one (in the Netherlands) a combination of public and private insurers (freely chosen by citizens) and contracting with private providers who are paid by a combination of per capita and per visit, while co-payments do not exist [19-22]. Last, one system (the US) is insurance based, but neither public nor compulsory, with insurers and providers being private (and providers being paid either from insurers or out-of-pocket), while government schemes for vulnerable groups (elderly, poor, Indians, veterans etc.) such as Medicare and Medicaid also exist. Also, because of the adoption of the Affordable Care Act in 2010, primary care and public health receive increased funding, while quality and expenditures are addressed through a range of measures. Case-mix methods are widely used, especially in Medicare, worth mentioning the Ambulatory Payment Classifications (APCs) and the Ambulatory Patient Groups (APGs) [23,24].

To recapitulate, examples can be drawn from eight healthcare systems for financing and providing of healthcare and PHC in particular. On a more policy oriented target, the potentials for cost reduction have to be quickly realized by redefining the insurance packages that can be provided from public funds as the level of commitment and solidarity to be given by our society. A new strategy that will fix health sector should be taken into account. Integrated Practice Units (IPU), outcome and cost evaluation, geographical patients’ needs, information technology are the starting points [25].

### b. Overview and analysis of Greek Healthcare focusing upon the EOPYY and PHC

Evolution of healthcare provision in Greece follows the country’s (turbulent up to 1974) social and political history with legislative tug-of-wars, drawbacks, expansions and collapses, whereas the dominant feature was the existence of many occupation-based and state-supported social and health insurance funds, usually compulsory, but leaving ample room for the private sector. A turning point was in 1983 the establishing of the Greek National Health Service by L 1397/83, which clearly stated state responsibility, and funding to government healthcare services that were of equal access and free at the point of delivery, whereas the pre-existing insurance funds remained. It should be however noted that despite a large number of existing funds purchasing health services, by early 2010s we can refer to IKA for private sector salaried employees, OPAD for state employees, OAEE for the self-employed and OGA for farmers and people living in small population rural areas. It is estimated these four combined covered approximately 90% of the population. Establishment of EOPYY by L 3918/11 that was approved by Parliament on February 11^th^ started operating on 1^st^ January 2012 as preparatory measures were needed, is a significant step in the said evolutionary process. This step was necessitated inter alia by the pressing fiscal, financial and of course funding constraints caused crisis and memorandum and its implementation overseen by the three lenders or else so called ‘Troika’. Further steps were taken under the Law 4238/14 making organisational and structural changes, such as the separation of the EOPYY (IKA) polyclinics from EOPYY and their affiliation with the NHS health centers, under Regional Health Authorities (RHAs), accompanied by setting personnel issues (allegedly partly related to the ‘memorandum’ and expenditure cuts through putting staff on probation before re-employing them under new conditions), but not significantly altering funding or accessibility. As this article wishes to focus on accessibility and funding of services, the next pages will continue concentrating on EOPYY and the arrangements of L 3918/11.

Returning to the main point of interest, it can therefore be claimed that currently in a nutshell the Greek health system is a mixture of three main components [26,1]:

1. A tax-based national health system (NHS) that is responsible for public hospitals and health centres in the rural areas (now in the urban areas too).
2. An extensive network of polyclinics belonging to insurance funds (mainly IKA), financed by insurance contributions paid by employees and employers. These units are mainly located in urban areas, covering more than 50% of the population. Their control and management was recently extorted from EOPYY and transferred to RHAs (unfortunately reducing their utilisation).
3. A private insurance system (fairly small and mainly consisting of supplementary insurance) and a private delivery system, consisting of private hospitals, diagnostic centres and private physicians, most of which have contracts with EOPYY (who in turn can not afford to finance all now).

Examining EOPYY in more detail, it can be observed that it runs its own healthcare delivery units in urban areas, covering more than 50% of the population, with about 25% remaining in rural areas. Actually, these units previously belonged to IKA and they were transferred to EOPYY as a key term of the merger.

Further to the analysis of the purchasers, the article is now turning the overview’s focal point to the point of the supply of PHC services. In rural areas (approximately 30% of the population) PHC is provided mainly by the NHS health centres, whereas in urban areas (70% of population) is mainly provided by the outpatient departments of public/private hospitals, EOPYY’s units and self-employed health professionals, whilst there is no integration of the different PHC services. Workforce is unbalanced, with too many physicians (many specialists and few GPs) and a lack of nurses, health visitors and other health professionals. In Greece, GPs account for 5% of total physicians (EU average being 25%). Greece has the largest number of physicians among the member states of the OECD and almost twice the average of the member states of the EU (3.3/1,000 inhabitants) with 6.1 physicians/1.000 inhabitants [27]. This absence of GPs as a first point of contact to health services favors an uncontrolled provision of medical examinations and diagnostic tests, whereas contribution of these examinations and tests to morbidity reduction or health status improvement is uncertain.

Regarding purchasing of services and remuneration of providers, once (EOPYY) was established, compulsory health insurance turned into a peculiar monopsony, as it is the sole purchaser of health services covering over 98% of the insured population, while prior to L 4238/14 used to be a PHC provider at the same time. This leaves little room for maneuver for providers (who can find no many other potential buyers of their services), and for other smaller insurers (who are dwarfed once compared with the organization) thus the use of the term monopsony. Due to the concurrent existence of numerous public insurance funds that were (each) contracting individually with providers, EOPYY’s establishment is undoubtedly the most promising reform of the last decades in Greek health insurance. However its performance in 2012–2013 doesn’t seem to have met the expectations of Greek society, Greek state as well as Troika yet. The organization is engaged in a vicious circle of deficits that characterize the domestic social insurance system in general [2], caused by a multiple of reasons such as the pre-existing shortfalls of the funds that were bequeathed to EOPYY, funding problems due to the crisis (unemployment, undeclared work, inability of employers or self-employed to pay contributions), topped-up by unregulated expenses and supplier induced demand. So far, EOPYY has just created successive deficits enforced by the reasons set briefly above.

Regarding the reimbursement system, self-employed professionals enjoy only fee-for-service payments, excluding physicians who earn additional income per visit. Physicians are reimbursed for a maximum number of visits per month; nevertheless it is not enough to constrict induced demand. On the other hand, physicians of the NHS and the EOPYY are salaried regardless their specialty. Another feature of the Greek health insurance market is the growing patient’s charges, which have either the form of fixed percentage rates on the total cost (see Table 1) or the form of a flat co-payment of €5 per visit to health centers and outpatient clinics.

**Table 1:** **Co-insurance rates**

**Categories of goods and services**	**Patient’s charges**
Preventive medicine	0%
Laboratory tests	15%
Health consumable materials	25%
Costed medical procedures	20% or 45%
Physiotherapy	0% (annual ceiling)
Speech therapy	0% (monthly ceiling)
Psychotherapy	0% (monthly ceiling)
Additional care and therapeutics	25%
Nursing in foreign public or private hospitals	5% or 10%
Pharmaceuticals	0-25%

Source: EOPYY [28].

Moreover, there are large geographical inequalities. Lack of equity of access to healthcare has been long described as a fundamental problem of Greek healthcare. However, equity demands redesigning PHC into an integrated model. The integration of primary care providers, the establishment of a multi-disciplinary team and the legislation for the family physician institution seem to be essential for continuous and efficient healthcare [29].

As mentioned in the introduction and now on set by the relevant legislation (i.e. Law 4238/14) Primary Health Care (PHC) is the level of a health system that provides entry into the system itself for all new needs and problems, providing person-focused care over time [30]. PHC is now well understood as the basis for rational health systems. The provision of PHC incorporates a set of attributes and characteristics: “First-contact” care; Continuous (ongoing) care; Coordinated care; Comprehensive care [31]. On the other hand, it should be mentioned that there is a large heterogeneity internationally and especially in Europe regarding PHC both as providers and spectrum of provision are under question. In most cases, the core of PHC is the family physician [32].

### c. Cost accounting under APGs principles

The costing process was tested in NHS health centers and included three steps:

I. **Encoding of APGs (‘OEPFYs’ in Greek)**. The most ordinary incidents of health centers were grouped into 15 categories that are homogeneous in terms of patient’s condition and required medical procedure. Evidently, each group is expected to include incidents of similar cost.

The 15 categories mentioned above are:

1. ***Emergency incident management*** (e.g. childbirth, acute myocardial infarction, acute chronic obstructive pulmonary disease ‘COPD’ exacerbation): Diagnostic, therapeutic interventions, prescription, referral, medical evacuation, are all included.
2. ***Management of acute (not emergency) disease*** (e.g. trauma fracture, respiratory infection, migraine crisis): Diagnostic, therapeutic interventions, prescription, referral, are all included.
3. ***Review of acute or emergency incident***: Diagnostic, therapeutic interventions, prescription, referral, are all included.
4. ***Physical health assessment*** (e.g. health certificate, first examination in a clinic for chronic diseases): Routine laboratory test, advanced preventive, diagnostic and therapeutic interventions are not included.
5. ***Partial physical health – chronic diseases review*** (e.g. constipation, osteoporosis, depression, glaucoma, and other vision problems): Routine laboratory test, advanced preventive, diagnostic and therapeutic interventions are not included.
6. ***Regular health check-up*** (e.g. natal care, monitoring infant and child development): Routine laboratory test is not included.
7. ***Laboratory exams*** without clinical examination (the same for categories 8–11 and 15).
8. ***Prescription*** (medicines and exams).
9. ***Diagnostic or therapeutic intervention*** (e.g. blood pressure and glucose measurement, injection therapy, wound care).
10. ***Advanced diagnostic or therapeutic intervention*** (e.g. nevus removal, ultrasound).
11. ***Children and adults vaccination***.
12. ***Primary prevention*** (e.g. tips for quitting smoking, contraception, alcohol, diet, sexually transmissible disease, accidents): Routine laboratory test is not included.
13. ***Advanced preventive intervention*** (e.g. ‘Pap’ test) without a total clinical examination.
14. ***Special assistance and support*** (e.g. physiotherapy, rehabilitation, psychotherapy, social support, terminal care) by a physician or other healthcare professional.
15. ***Addressing administrative issues*** (e.g. receiving exam answers, taking referrals, correcting prescriptions’ error).
16. **Matching APGs with the ICPC-2 coding**. International Classification of Primary Care consists of 17 chapters, each divided into 7 components concerning process codes, symptoms and complaints, infections, neoplasms, injuries, congenital anomalies and other diagnoses [33-35].
17. **Development of a cost sheet**. This includes a) staff classification in medical, nursing, administrative and other staff; b) calculation of the standard unit labor cost (per staff category) c) insight upon contribution rate per category of incidents (per staff category) and d) determination of other direct cost (materials, drugs and examinations the prices of which was mainly obtained from Health Procurement Commission’s (EPY’s) Observatory, as well as determination of indirect cost (overheads) absorbed on a man-hours basis.

The costing process was completed in June 2013 in the health centers of Vari (prefecture of Attica) and Michaniona (prefecture of Thessaloniki) taking into account 79 patient incidents.

## Results

A prime aim of the survey resulting in the current article was associated with the upgrading of the gatekeeping role of GPs, working as group practices. Specifically concerning reimbursement of contracted first contact physicians (GPs and family pediatricians) could be established at the rate of at least €20 per capita (with a registered population between 1,000 and 2,000). Additional payments can also be established as an incentive for the simultaneous management of multiple health problems in the same patient episode of care and the necessary preventive medical activities (health education, management of major risk factors, etc.). These additional payments imply a pay-for-performance scheme similar to this applied by UNCAM (France) through CAPIs [18].

Additional, on EOPYY’s charge, payments can be established in case of (i) screening (€5 per case/screening), (ii) covering elderly people over 65 (€5 per capita/patient), (iii) chronically ill population care (€5 per capita/patient), and (iv) home visits (€5 per visit, maximum 3 visits per year per each registered beneficiary). Moreover, first contact physicians could receive a type of overtime allowance for 24-hour services provision to beneficiaries. Should such a system be established, a co-payment of 5 Euros (in patient’s charge) for each visit to specialists without a referral from a family physician or pediatrician becomes essential. In other words, beneficiaries could visit specialists without charge only after referral by physicians of first contact. Returning our focus upon contracted specialists and other professionals, they will continue to be reimbursed on a fee-for-service basis, specifying visiting hours and/or a higher upper ceiling of visits per month (up to 300 visits), but an adjustment to regional global budgets inspired from the Allgemeine Ortskrankenkasse (General Local Health Insurance Fund) (or else AOK) is necessary in a system totally summing up to approximately one billion euro. As a result specialists’ payments depend not only on the volume of services they provide but also on the global budget of each health region. Another high co-payment (about 30% of the reimbursable cost) is needed for laboratory and radio diagnostic tests or visits to health professionals without a referral from a contracted physician. A same purpose but lower (20% of the reimbursable cost) co-payment is charged in Austria [15], however Greece faces significant problems of the so called “induced demand”, mainly due to doctors’ oversupply.

GPs of health centres, regional offices and EOPYY’s polyclinics can maintain their fixed salary, but they will also be able to sign contracts with EOPYY. These contracts will ensure them a motive of €10 per capita over 1,000 people. Moreover, they are beneficiaries of additional payments like those of self-employed GPs. Structural and administrative changes set by Law 4238/2014 referring to transfer of responsibility and authority to RHAs including the setting and function of mobile health units, administration of EOPYY, and mainly issues relating to staff salaries, directly influence implementation of the said proposals. However the underlying solutions suggested after the method of benchmarking health systems in various countries and setting them vis-à-vis problems of the Greek system (viz. per capita and additional payments, salaries and extra contracts etc.) remain as policy proposals for an ameliorated and with more efficient expenditure healthcare delivery system regardless of its form in Administrative Law.

**Figure 1:**
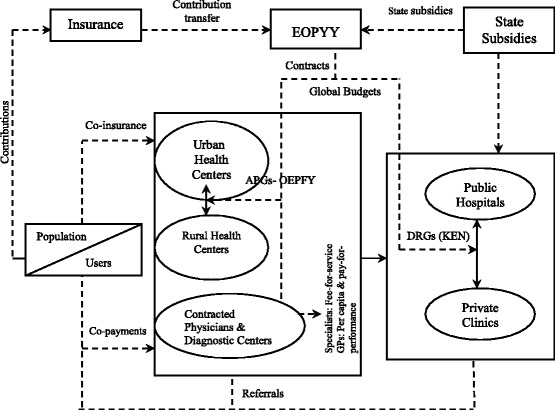
**Flows of proposed Health Provision and Financing in Greece.**

Taking into account the best practices from developed health systems and funds mentioned above vis-a-vis the situation in Greece, our proposal is extended to a new model of provision (Figure 1) whereby PHC providers are interconnected and funded by a mixture of users’ charges and formal payments (as described above), whilst EOPYY is the unique pool of state subsidies and contributions intending for primary and secondary care financing.

Moreover, Table 2 represents the proposed distribution of NHS PHC units and staff (hereinafter Rural Health Centers) merged with EOPYY PHC units and staff (hereinafter NHS Urban Health centres) according to population and other criteria. In the same context, Table 3 represents the proposed (re-)distribution of self-employed physicians contracted with EOPYY in order to create an intergraded PHC network and fade out geographical and specialty inequalities. This implies either signing new or breaking existing contracts. It’s also obvious that GPs and pediatricians should spearhead the reformed provision model.

**Table 2:** **Proposed distribution of the PHC units and staff**

**Health regions** ^**a**^	**Health centers (NHS + EOPYY)**	**GPs**	**Laboratory physicians**	**Other specialists** ^**b**^	**Nursing – Health staff**	**Admini-strative & other staff**	**Total**
**1** ^**st**^	90	270	270	1,350	1,430	1,000	4,320
**2** ^**nd**^	60	120	120	600	630	450	1,920
**3** ^**rd**^	45	120	120	600	630	440	1,910
**4** ^**th**^	50	130	130	650	690	480	2,080
**5** ^**th**^	55	140	140	700	740	515	2,235
**6** ^**th**^	80	170	170	850	900	630	2,720
**7** ^**th**^	20	50	50	250	280	185	815
**Total**	**400**	**1,000**	**1,000**	**5,000**	**5,300**	**3,700**	**16,000**

^a^Allocation to health regions on a population basis catchment, ^b^among them 1000 pediatricians & more GPs.

**Table 3:** **Proposed distribution of contracted with EOPYY physicians**

**Specialty/Health regions** ^**a**^	**1** ^**st**^	**2** ^**nd**^	**3** ^**rd**^	**4** ^**th**^	**5** ^**th**^	**6** ^**th**^	**7** ^**th**^	**Total (proposal)**	**Total (current)**	**Deviation** ^**b**^
GPs	280	124	180	74	124	163	55	**1,000 (14.3%)**	420 (7.7%)	+580
Pathologists (internal medicine)	336	149	216	89	149	195	66	**1,200 (17.1%)**	1,246 (22.7%)	−46
Cardiologists	224	99	144	59	99	131	44	**800 (11.4%)**	842 (15.3%)	−42
Pediatricians	280	124	180	74	124	163	55	**1,000 (14.3%)**	206 (3.8%)	+794
Obstetricians/gynecologists	168	74	108	44	74	99	33	**600 (8.6%)**	569 (10.4%)	+31
Orthopedicians	112	50	72	30	50	64	22	**400 (5.7%)**	383 (7.0%)	+17
Other physicians (pathologists)	420	186	270	111	186	245	82	**1,500 (21.4%)**	1,650 (30.1%)	−150
Other physicians (surgeons)	140	62	90	37	62	82	27	**500 (7.1%)**	170 (3.1%)	+330
**Total**	**1,960**	**868**	**1,260**	**518**	**868**	**1,142**	**384**	**7,000 (100.0%)**	**5,486 (100.0%)**	**+1,514**
**Per 1,000 population**	**0.64**								**0.50**	**+0.14**

^a^Allocation to health regions mainly on a catchment population basis.

^b^“+”: new contracts “-”: breaking existing contracts.

Continuing on the argument set above, and given the economic predicament and the immediate need to stop the regressive and ultimately ineffective and inefficient funding, a necessary and urgent reform is the transition to an alternative system of both urban and rural health centers’ reimbursement. This alternative system should be based on APGs. The costing of the so called OEPFYs, as presented on Table 4, suggests an average cost 20.89 euros per incident. The highest cost was noted in the category of direct cost (euro 12.67), and especially that of laboratory tests (euro 8.29). It is therefore obvious that in all APGs recorded direct cost performs the main contribution to the total cost per incident (by 60.64%) coupled by direct labor cost (31.36%). Indirect cost in its turn seems to occupy only 8% of the total. The contribution of highly expensive laboratory tests (approximately 65% of direct cost) is primarily significant. It is not surprising therefore, that the patient incidents, which usually include laboratory tests (see 4, 5 & 7 category), are the APGs with the highest cost.

**Table 4:** **Mean average per APGs in the Greek NHS Primary Health Care units**

**APGs (OEPFY)**	**Cost categories**										**Total (1) + (2) + (3) + (4)**
	**No of incidents**	**Direct labor (1)**	**Drugs/ medicines (2.1)**	**Sanitary materials (2.2)**	**Direct materials (2) = (2.1) + (2.2)**	**Laboratory tests (3.1)**	**Radiodiagnostic tests (3.2)**	**Dental tests (3.3)**	**Tests (3) = (3.1) + (3.2) + (3.3)**	**Indirect cost/Overheads (4)**	
1	9	10.3114	1.1302	1.1665	2.2967	2.0544	4.7333	0.0000	6.7878	2.4044	**21.8003**
2	10	4.5590	1.5738	0.6288	2.2026	0.1760	2.5000	0.0000	2.6760	1.1949	**10.6325**
3	5	3.2576	0.9072	0.0245	0.9317	0.0000	2.5700	0.0000	2.5700	1.0062	**7.7654**
4	6	5.4293	0.0000	0.0107	0.0107	33.3450	6.7500	0.0000	40.0950	1.4651	**47.0001**
5	9	6.3432	0.3161	0.0071	0.3233	23.8467	2.3278	0.0000	26.1744	1.5633	**34.4042**
6	6	6.5905	2.4017	0.0557	2.4573	0.7733	0.0000	13.0983	13.8717	1.7269	**24.6463**
7	6	18.9890	0.0000	3.7333	3.7333	32.5283	0.0000	0.0000	32.5283	4.2622	**59.5128**
8	3	3.5489	0.0000	0.0000	0.0000	0.0000	0.0000	0.0000	0.0000	1.0377	**4.5866**
9	6	1.8398	0.0000	0.3246	0.3246	0.0000	0.0000	0.0000	0.0000	0.7279	**2.8923**
10	3	8.7933	0.2746	2.6278	2.9023	0.0000	1.3500	0.0000	1.3500	1.8850	**14.9306**
11	4	3.6526	0.0000	0.2089	0.2089	0.0000	0.0000	0.0000	0.0000	1.1925	**5.0540**
12	4	7.2376	0.0000	0.0000	0.0000	0.0000	0.0000	0.0000	0.0000	2.0293	**9.2668**
13	3	4.3651	0.0947	7.4067	7.5013	6.6600	0.0000	0.0000	6.6600	1.4033	**19.9297**
14	1	3.7879	0.0000	0.0000	0.0000	0.0000	0.0000	0.0000	0.0000	1.2671	**5.0550**
15	4	2.8003	0.0000	0.0000	0.0000	0.0000	0.0000	0.0000	0.0000	0.8092	**3.6096**
**Total (weighted average)**	**79**	**6.5510**	**0.6178**	**0.9197**	**1.5375**	**8.2877**	**1.8475**	**0.9948**	**11.1300**	**1.6728**	**20.8914**
**% of total**		**31.36%**	**2.96%**	**4.40%**	**7.36%**	**39.67%**	**8.84%**	**4.76%**	**53.28%**	**8.01%**	**100.00%**
**Median**		**4.8701**	**0.0000**	**0.0160**	**0.1747**	**0.0000**	**0.0000**	**0.0000**	**0.0000**	**1.3220**	**11.5829**
**Minimum**		**1.1364**	**0.0000**	**0.0000**	**0.0000**	**0.0000**	**0.0000**	**0.0000**	**0.0000**	**0.6183**	**2.1894**
**Maximum**		**36.9318**	**10.6100**	**13.5000**	**13.6420**	**71.3400**	**12.8500**	**42.3000**	**75.3900**	**8.3512**	**122.2230**

Finally, the proposed types of reimbursement are related to the (minimum) global (at least) budget for all categories of PHC providers (Table 5), regarding the results of each health unit, sorted by categories of cases (patients). Indispensable condition for the patient classification into APGs is the existence of a developed system at the base of International Classification of Diseases/International Classification of Primary Care (ICD-10/ICPC-2) and Current Procedural Terminology (CPT-4), as well as the necessary data extraction from an extensive database. However, the availability of such a database is extremely limited because of the absence of electronic patient records, and of updated and reliable healthcare billing system. As hinted above, the proposed reimbursement method constitutes a conjunction between German global budgets and US Medicare case-mix models.

**Table 5:** **PHC Global Budget of EOPYY per region and category of contracted group of providers (million Euros – at least)**

	**Health regions**							
**Provider**	**1** ^**st**^	**2** ^**nd**^	**3** ^**rd**^	**4** ^**th**^	**5** ^**th**^	**6** ^**th**^	**7** ^**th**^	**Total**
Health centers & regional offices	20	35	25	25	35	45	15	**200**
EOPYY’s units	85	15	30	30	35	40	15	**250**
Contracted GPs	30	10	10	10	15	20	5	**100**
Contracted specialists	50	15	15	15	20	30	5	**150**
Diagnostic laboratories	69	20	35	35	36	37	18	**250**
Others	16	5	5	5	8	9	2	**50**
**Total**	**270**	**100**	**120**	**120**	**149**	**181**	**60**	**1,000**
Catchment population (in millions)	3.00	1.05	1.30	1.30	1.60	1.90	0.65	10.80

Benchmarking other health systems has resulted to proposals for restructuring and setting new schemes of providers’ remuneration by EOPYY, also taking into account recent legislative developments. A discussion presenting difficulties faced during our survey follows in next section Discussion.

## Discussion

Any self-critical discussion of proposals should commence with limitations encountered during survey and then turn to an overall approach to question of how to set policy proposals.

### a. Limitations

A key hindrance to the survey presented was the failure from the official agency of the EOPYY to provide us with the necessary and available information and data. This lack of the necessary data of our research has prompted us to present annual estimates of medical visits and diagnostic tests and their cost.

Thus, the total medical visits and diagnostic tests per annum have been estimated to 50 million visits alongside 50 million tests, with a total cost of 650 million euro for the medical visits and 250 million euros for the diagnostic tests (100 million remaining for other health professionals or/and for corrections to the above). Furthermore, the average examination cost of each of the total 15 APGs categories was calculated and multiplied by the estimated number of tests for each group of providers (health centres, outpatient departments of NHS hospitals, EOPYY polyclinics and affiliated physicians with EOPYY) as to assist in cross checking and to inform us over cost estimates by provider.

Some additional limitations of the study were the fact that the labour-hours required for each incident were recorded through personal interviews with employees of the two main health centres, mainly with physicians. The fluctuation of the relative cost, can be attributed to the variety of (the same active substance) medicines that are used by the health centres, and to the fact that the indirect cost varies among health centres, despite salaries and medicine prices being centrally controlled.

Additionally legislative changes occurred during the final stages of writing this paper (L4238) have set a new frame, which however does not alter much the key ideas concerning accessibility and funding as the said Law relates rather to administration and structure.

### b. The setting of policy proposals

Health systems have to learn and benefit from each other via adopting and adapting methods and practices in financing, delivering and remunerating healthcare services, not to mention patients’ flow between primary and secondary (or indeed tertiary) healthcare services. All four depend upon decisions, choices and habits of the past, alongside social practices and culture relating to healthcare and physician-patient or even physician-society inter-connecting bonds and relations for that matter. This is therefore why instead of a system of copying and almost automatic implementation, a process of selective adoption and of critical, flexible and suitable adaptation is overall chosen and suggested, as well as followed. In short our proposals related to the betterment of the Greek system, are based upon selective adoption and critically appraised adaptation. Redesigning PHC should be led to improve values in order to meet patients’ needs towards groups of patients and team-based professionals and services [36]. Payments would be modified to succeed an effective relation between them and third party payer, under productivity and quality standards. This is what our study tried indicatively to solve in Greece.

Taking into account the restrictions mentioned above and the future abolition of them, we hope to lay the foundations for more detailed Greek APGs (OEPFY) aiming at modernizing the financing (reimbursement) system of units of PHC. Our proposal is to enhance the further costing per individual visit-incident, based on the APGs, combined by the matching of the ICPC-2 coding.

This is one of the tasks of final section Conclusions on conclusions that examines how Greek healthcare provision can benefit -using international benchmarks- within a restrictive financial and fiscal framework and environment.

## Conclusions

Greek healthcare provision (and moreover primary healthcare provision) can benefit by an “a’ la cart” picking up of solutions internationally implemented and their subsequent adaptation to the now existing national system taking current crisis into account. Such an approach can easily commence with ‘negative selection’ or else exclusion of the American and Dutch systems as they rely too much upon private insurance and provision (with the American currently trying to use the Dutch as an example for its expansion). Turning to what can be used as examples in organization structure, the English, Swedish and Spanish systems of regional administration can be followed (excluding the Swedish idea of regional self funding as Greece has not such an experience in regional taxation on the one hand, and has important differences in wealth and per capita wealth and income between regions on the other). Such an administrative structure can be followed and indeed assist a better positioning of services by overlooking staffing of Health Centers, giving motives to physicians and other personnel (nursing staff, auxiliaries) as to reduce patient overcrowding at urban areas (mainly regional hospitals, and predominantly Athens and Thessaloniki). This latter strengthening of services at the localities can lead to stronger ‘gatekeeping’ by PHC practitioners, and thus ultimately assist in overall cost reduction. Adopting the German idea of fee paying for secondary care if no referral from primary care practitioner or service is presented, but adapting it to a lower level e.g. 5 instead of 10 euro can also assist such a goal. In this sense, a contract can be drawn (and offered) to PHC group practitioners/physicians of first instance requiring compulsory coverage of a basic range of medical services (coupled by laboratory assistance and ability for secondary care referrals) towards to specific group of patients.

On the other hand, the recently unified fund (EOPYY) could be primarily a sole purchaser of services setting by contracts the rules of the game for providers and assessing their performance with the ability to strike of its register unsatisfactory ones and attract promising individuals. Last but not least, EOPYY funding can be as currently drawn from a combination of national insurance contributions and general budget assistance (direct, property and indirect taxes), as well as EOPYY management should be taken more attention to be more independent and effective.

Greek PHC needs over a billion Euro (public or/and social insurance money) in order to achieve universal coverage, taken into account the private money too. It is enormously difficult to ensure this amount especially under the ongoing crisis. Our core approach includes saving financial resources wasted on induced demand practices, unnecessary and irrationally priced care. EOPYY should distribute this money geographically (global budget) per service contracted (NHS and Private GPs, specialists and others). Our research covered this proposal and more over units’ and physicians’ remuneration. This could be set upon a multiple of choices ranging from salary for National Health Service Health Centers (and EOPYY transferred to NHS) staff, plus incentives proposed, and going on the per capita payments (following a combination of the English workload weighted system and the German regional negotiations system) for self-employed GPs, and fee for service (coupled by Ambulatory Patients Groups cost estimate) for specialists, on a Greek but rational way.

## Abbreviations

AOK: Allgemeine Ortskrankenkasse (General Local Health Insurance Fund, Germany)
APG(s): Ambulatory Patients Group(s)
CAPIs: Contrats d’ amelioration des pratiques individuelle) (France)
CHF: Central Health Fund (Germany)
COPD: Chronic obstructive pulmonary disease
CPT-4: Current Procedural Terminology
ECB: European Central Bank
EOPYY: National Organization for Healthcare Provision (Greece)
EPY: Health Procurement Commission (Greece)
EU: European Union
GP(s): General practitioner(s)
HMO(s): Health Maintenance Organization(s) (United States)
ICD-10: International Classification of Diseases
ICPC-2: International Classification of Primary Care
IMF: International Monetary Fund
IPU(s): Integrated Practice Unit(s)
NHS: National Health Service
OECD: Organisation of Economic Co-operation and Development
OEPFY: (Greek) APGs
PHC: Primary Health Care
PPO(s): Preferred Provider Organization(s) (Unite States)
UNCAM: National Union of Health Insurance Funds (France)

## References

[1] Economou C (2010). Greece: health system review. Health Syst Transit.

[2] Niakas D (2013). Greek economic crisis and health care reforms: correcting the wrong prescription. Int J Health Serv.

[3] Polyzos N, Kastanioti C, Theodorou M, Karakolias S, Mama K, Thireos E, Polizoidis P, Skamnakis C, Tsairidis H, Dikaios C (2013). Study on reimbursement system of public and private primary health care units contracted with EOPYY.

[4] Chassard Y, Quintin O (1992). Social protection in the European Community: towards a convergence of policies. Int Soc Sec Rev.

[5] Boyle S (2011). United Kingdom (England): heath system review. Health Syst Transit.

[6] WHO Regional Office for Europe (2011). European Health for All database [HFA-DB].

[7] Clasen J (2011). Worlds of Welfare? British and German Social Policy in the 21st Century.

[8] Anell A (2011). Choice and privatisation in Swedish primary care. Health Econ Policy Law.

[9] Swedish Association of Local Authorities and Regions (SALAR) (2012). Quality and efficiency in Swedish Health Care – Regional Comparisons 2012.

[10] Anell A, Glenngard A, Merkur S (2012). Sweden: health system review. Health Syst Transit.

[11] García-Armesto S, Abadía-Taira M, Durán A, Hernández-Quevedo C, Bernal-Delgado E (2010). Spain: health system review. Health Syst Transit.

[12] Health Information Institute (2010). National Health System of Spain, 2010.

[13] Busse R, Riesberg A (2004). Health care systems in transition: Germany.

[14] Göpffartha D, Henkeb K (2013). The German central health fund: Recent developments in health care financing in Germany. Health Policy.

[15] Hofmarcher M, Rack H-M (2006). Austria: health system review. Health Syst Transit.

[16] Franc C, Polton D (2006). New governance arrangements for French health insurance. Eurohealth.

[17] Bellanger M, Cherilova V, Paris V (2005). The “Health Benefit Basket” in France. Eur J Health Econ.

[18] Chevreul K, Durand-Zaleski I, Bahrami S, Hernández-Quevedo C, Mladovsky P (2010). France: health system review. Health Syst Transit.

[19] Maarse Η, Bartholomée Y (2007). A public–private analysis of the new Dutch health insurance system. Eur J Health Econ.

[20] Vaillancourt Rosenau P, Lako C (2008). An experiment with regulated competition and individual mandates for universal health care: the new Dutch health insurance system. J Health Polit.

[21] Van der Feltz-Cornelis C, Knispel A, Elfeddali I (2009). Treatment of mental disorder in the primary care setting in the Netherlands in the light of the new reimbursement system: a challenge?. Int J Integr Care.

[22] Schäfer W, Kroneman M, Boerma W, Van den Berg M, Westert G, Devillé W, Ginneken E (2010). The Netherlands: health system review. Health Syst Transit.

[23] U.S. Department of Health and Human Services (2012). SCHIP Overview.

[24] Rice T, Rosenau P, Unruh L, Barnes A, Saltman R, van Ginneken E (2013). United States of America: health system review. Health Syst Transit.

[25] Porter M, Lee T (2013). The Strategy that will fix Health Care. Harv Bus Rev.

[26] Mossialos E, Allin S, Davaki K (2005). Analysing the Greek health system: a tale of fragmentation and inertia. Health Econ.

[27] OECD: *OECD Health Statistics 2014 (database)*. http://stats.oecd.org/index.aspx?DataSetCode=HEALTH_STAT

[28] EOPYY (2013). Instructions to Beneficiaries.

[29] Lionis C, Symvoulakis E, Markaki A, Vardavas C, Papadakaki M, Daniilidou N, Souliotis K, Kyriopoulos I (2009). Integrated primary health care in Greece, a missing issue in the current health policy agenda: A systematic review. Int J Integr Care.

[30] Starfield B (1994). Is primary care essential?. Lancet.

[31] Starfield B (1979). Measuring the attainment of primary care. J Med Educ.

[32] Boerma W, Saltman R, Rico A, Wienke G, Boerma W (2006). Coordination and integration in European primary care. Primary care in the driver’s seat? Organizational reform in European primary care.

[33] Goldfield N, Averill R, Eisenhandler J, Grant T (2008). Ambulatory Patient Groups, Version 3.0 – A classification system for payment of ambulatory visits. J Ambul Care Manage.

[34] Averill R, Goldfield N (1993). Design of a prospective payment patient classification system for ambulatory care. Health Care Financ Rev.

[35] Kapriel M (1994). Using patient classification systems to identify ambulatory care costs. Healthc Financ Manage.

[36] Porter M, Pado E, Lee T (2013). Redesigning primary care: a strategic vision to improve value by organizing around patients’ needs. Health Aff.

